# Nitric Oxide in the Pathogenesis and Treatment of Tuberculosis

**DOI:** 10.3389/fmicb.2017.02008

**Published:** 2017-10-12

**Authors:** Hamidreza Jamaati, Esmaeil Mortaz, Zeinab Pajouhi, Gert Folkerts, Mehrnaz Movassaghi, Milad Moloudizargari, Ian M. Adcock, Johan Garssen

**Affiliations:** ^1^Chronic Respiratory Research Center, National Research Institute of Tuberculosis and Lung Diseases, Shahid Beheshti University of Medical Sciences, Tehran, Iran; ^2^Clinical Tuberculosis and Epidemiology Research Center, National Research Institute of Tuberculosis and Lung Diseases, Shahid Beheshti University of Medical Sciences, Tehran, Iran; ^3^Department of Immunology, School of Medicine, Shahid Beheshti University of Medical Sciences, Tehran, Iran; ^4^Division of Pharmacology, Faculty of Science, Utrecht Institute for Pharmaceutical Sciences, Utrecht University, Utrecht, Netherlands; ^5^Cell and Molecular Biology Group, Airways Disease Section, Faculty of Medicine, National Heart and Lung Institute, Imperial College London, London, United Kingdom; ^6^Priority Research Centre for Asthma and Respiratory Disease, Hunter Medical Research Institute, University of Newcastle, Newcastle, NSW, Australia; ^7^Nutricia Research Centre for Specialized Nutrition, Utrecht, Netherlands

**Keywords:** nitric oxide, non-tuberculous mycobacteria, Mycobacterium, macrophages, drug-resistance, nitric oxide donors

## Abstract

*Mycobacterium tuberculosis* (Mtb), the causative agent of tuberculosis (TB), is globally known as one of the most important human pathogens. Mtb is estimated to infect nearly one third of the world's population with many subjects having a latent infection. Thus, from an estimated 2 billion people infected with Mtb, less than 10% may develop symptomatic TB. This indicates that the host immune system may constrain pathogen replication in most infected individuals. On entering the lungs of the host, Mtb initially encounters resident alveolar macrophages which can engulf and subsequently eliminate intracellular microbes via a plethora of bactericidal mechanisms including the generation of free radicals such as reactive oxygen and nitrogen species. Nitric oxide (NO), a key anti-mycobacterial molecule, is detected in the exhaled breath of patients infected with Mtb. Recent knowledge regarding the regulatory role of NO in airway function and Mtb proliferation paves the way of exploiting the beneficial effects of this molecule for the treatment of airway diseases. Here, we discuss the importance of NO in the pathogenesis of TB, the diagnostic use of exhaled and urinary NO in Mtb infection and the potential of NO-based treatments.

## Introduction

The formation of the gaseous mediator nitric oxide (NO) from L-arginine by NO synthases (NOS) has been traditionally considered as the first-line defense against parasitic infections in all species including metazoans (Schmidt and Walter, 1994). The importance of NO production and its release in pathogen-directed defense mechanisms has been confirmed by the effect of NOS inhibitors (Boom, 1996; Flynn et al., 1998; Sciorati et al., 1999) and NOS knock-out mice on enhancing the severity of infection and of exacerbations *in-vivo* and *ex-vivo* (Cooper et al., 2000; Kuo et al., 2000). However, there is uncertainty regarding the magnitude of the response and which strains of bacilli are the most susceptible to NO-induced killing (Denis, 1991; Flesch and Kaufmann, 1991; Chan et al., 1992; Appelberg and Orme, 1993; Doi et al., 1993; Rhoades and Orme, 1997; Garbe et al., 1999).

**Figure 1:**
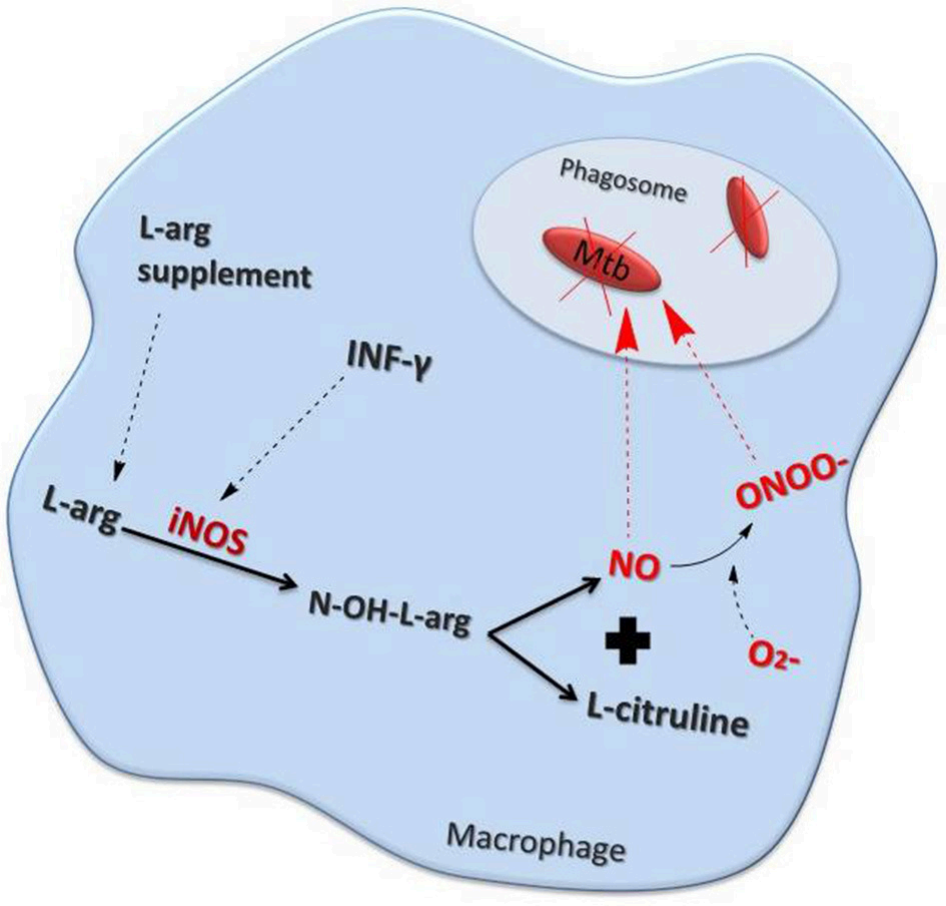
Nitric oxide (NO) production pathway inside a macrophage. Interferon (IFN)-γ as well as other inflammatory stimuli increase NO production by stimulating inducible nitric oxide synthase (iNOS). Elevated levels of the NO precursor, L-arginine (L-arg) also enhances NO production. NO may either act directly, or in combination with superoxide (∙O2–) to form peroxynitrite (ONOO∙), to kill mycobacteria (Mtb) within the phagosome.

NO also plays an important role in bacteriostatic and bactericidal processes as part of the host defense mechanisms against pulmonary infections (Flesch and Kaufmann, 1991; Appelberg and Orme, 1993). For example, inflammatory stimuli can enhance NO release via the up-regulation of the inducible form of NOS (iNOS or NOS2) within inflammatory macrophages (Liew and Cox, 1991; Nathan and Hibbs, 1991; Wang et al., 1998). NO is converted into highly Reactive Nitrogen Species (RNS) such as NO3– and NO2– within infected macrophages to drive bacterial death. The term, Reactive Oxygen Intermediates (ROI) refers to the reduction products of oxygen and include superoxide (∙O2), hydrogen peroxide (H2O2), and the hydroxyl radical (∙OH). These reactive products also form reactive conjugates with halides and amines, as well as with NO, giving rise to the production of peroxynitrite (ONOO–) (Nathan and Shiloh, 2000) (Figure 1).

The bactericidal effect of NO in human tissue macrophages may be direct or indirect via RNS (Rich et al., 1997; Scanga et al., 2001). However, there is evidence that the bactericidal effects of RNS may be an artifact of *in vitro* laboratory conditions as a reduced effect is seen under less harsh, more physiological conditions (Garbe et al., 1999). Bacillus Calmette–Guérin (BCG)-inoculated alveolar macrophages (AM) from patients with pulmonary fibrosis express increased levels of NOS2 protein and mRNA as well as peroxynitrite (Nozaki et al., 1997). Furthermore, human AM-induced killing of cytoplasmic BCG is attenuated by the NOS inhibitor NG-monomethyl-L-arginine monoacetate implicating both NO and peroxynitrite in this process (Nozaki et al., 1997). The importance of NO and an altered immune system in the control of tuberculosis (TB) infection was further shown with the altered levels of fractional exhaled NO (FeNO) and urinary NO metabolites reported in TB patients particularly those immunocompromised by human immunodeficiency virus (HIV) infection (Idh et al., 2008). These data highlight the critical role of NOS2 and of reactive nitrogen intermediates (RNI) in controlling mycobacterium bacilli infection of macrophages (Denis, 1991; Flesch and Kaufmann, 1991; Chan et al., 1992).

**Figure 2:**
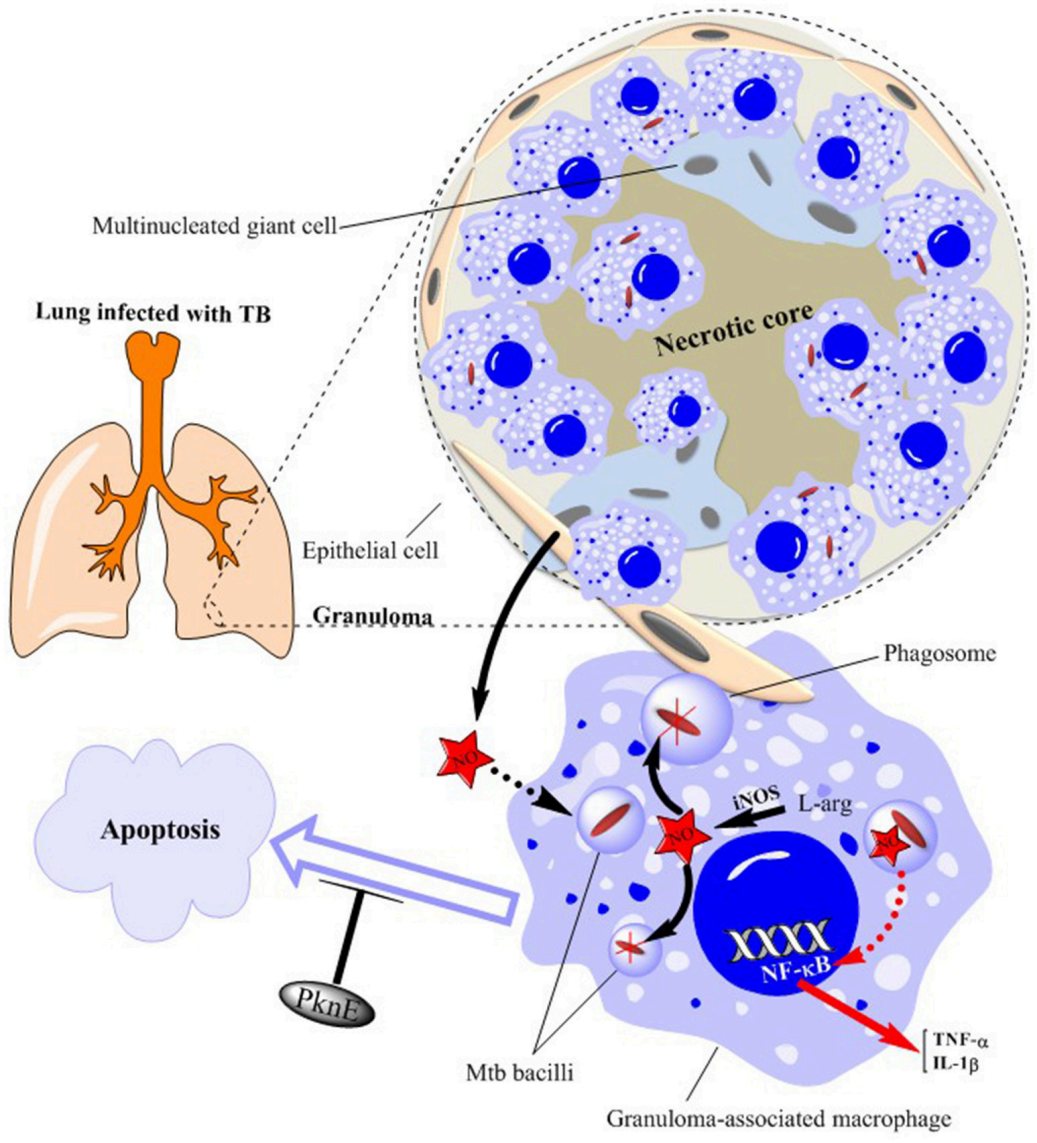
Schematic illustration of granuloma components in a TB-infected lung. Mtb bacilli are ingested by macrophages within the lung. This produces a profound inflammatory and immune response which ultimately leads to the formation of granulomas consisting of epithelioid and multinucleated giant cells. Mtb bacilli within the granuloma-associated macrophages are killed by nitric oxide (NO) generated from inducible NO synthase (iNOS)—see expanded macrophage. NO production also stimulates NF-κB activation leading to the production of inflammatory cytokines such as TNFα and IL-1β. Epithelial cells surrounding the granuloma further support bacterial killing by producing more amounts of NO. Mtb infection results in inhibition of macrophage apoptosis as a means of increasing its survival which is prevented by altered levels of the serine/threonine protein kinase, PknE. NO, nitric oxide; PknE, Protein Kinase E; Mtb, *Mycobacterium tuberculosis*.

Phagocytosis of *Mycobacterium tuberculosis* (Mtb) bacilli by mononuclear cells is the primary immunological mechanism in the face of TB infection (Edwards and Kirkpatrick, 1986; Rook et al., 1986; Wang et al., 2001). When exposed to Mtb, individuals who are healthy and immune-competent will mount an effective early and late immune response and do not develop the disease. In those who do develop disease, the initial response is mediated mainly by phagocytes while the late response is characterized by the action of CD4+ T-cells (Dunn and North, 1995). This ultimately leads to the formation of granulomas consisting of epithelioid and multinucleated giant cells (Kaufmann, 1993) (Figure 2). The severity of the disease is determined by phagocytic cells including polymorphonuclear cells (PMN), monocytes and AM (Aston et al., 1998). Indeed, the diagnosis of acute Mtb infection is often aided by the observation of abundant PMNs in the bronchoalveolar lavage (BAL) fluid (Zhang et al., 1995). Building on the above-mentioned *ex vivo* and *in vivo* data highlighting the importance of NO in TB infection and the altered levels of urinary and exhaled NO levels in infected patients, we review here the mechanisms by which NO regulates TB pathogenesis, the potential use of NO as a diagnostic of early infection and the future of NO-based therapeutic interventions.

## The role of NO and ONOO in anti-Mtb immunity

NO is an endogenous molecule produced at different sites throughout the body (Mikaili et al., 2014). This molecule is chemically active and is effective against a variety of pathogens including Mtb. Different mechanisms are used for killing Mtb *in vivo*, such as acidification of the phagosomes and phagosome-lysosome membrane fusion along with granzyme, granulysin, and perforin production (Lewinsohn et al., 1998; Stenger et al., 1998; Ernst et al., 2000; Serbina et al., 2000). These, together with ROI-mediated antimicrobial mechanisms, help in killing Mtb (Figure 1). The precise role of ROI in Mtb killing is difficult to accurately discern as peroxynitrite is ineffective in rodents and different strains of Mtb have differing sensitivity to NO (Cunningham-Bussel et al., 2013). However, studies in rodent cells may not give accurate insight into human disease as they generally produce greater quantities of NO compared to human cells although this may also relate to the culture conditions used (Cunningham-Bussel et al., 2013). It is important, therefore, that future studies investigating the role of NO and ROI in Mtb killing should be performed in primary human AMs in addition to experiments being performed *in vivo* (Yu et al., 1999; Chan et al., 2001a; Yang et al., 2009). In addition, the measurement of the intracellular survival of the bacilli should be undertaken (Yu et al., 1999). NO production is not only increased in macrophages but NOS2 mRNA expression and the production of NO is increased in A549 lung epithelial cells following infection with Mtb bacilli. A549 cells produced greater amounts of NO compared to macrophages which implicates the active involvement of lung epithelial cells in the anti-mycobacterial host defense process (Kwon et al., 1998).

The pivotal role of NO in protecting against infection by mycobacterial species is well-established. For example, NO production is the major determinant of macrophage resistance to *Mycobacterium bovis* (*M. bovis*)-induced infection, at least in comparison to apoptosis (Esquivel-Solís et al., 2013). Macrophages from cattle infected with, but protected against, *M. bovis* have a two-fold lower bacterial load and produce more NO than macrophages from cattle infected and not resistant to infection (Alcaraz-López et al., 2016). In addition the presence of inhibitors of arginine-dependent NO production results in a more rapid growth of *M. bovis* in infected cattle (Nozaki et al., 1997).

## Factors affecting NOS expression and NO production

The expression of the solute carrier family 11 member 1 (SLC11A1) gene determines the susceptibility or resistance to *in vitro* infection with the H37Rvt strain of Mtb. This is a consequence of the differential capability of resistant and susceptible macrophages to produce NO in response to Mtb (Arias et al., 1997). In addition, interferon (IFN)-γ and lipopolysaccharide treatment enhanced the expression of the arginine permease, MCAT2B, but this could not account for the observed increase in L-[^3^H] arginine uptake. Together this indicates that the activity of the L-arginine transporter(s) may also alter in response to macrophage activation (Peteroy-Kelly et al., 2001) (Figure 1).

NOS2 is not homogenously distributed within macrophages but is preferentially distributed in newly formed phagosomes following receptor-mediated uptake of latex beads opsonized with either complement products or IgG (Miller et al., 2004). However, the intraphagosomal NOS2 localization is not seen following infection with *M. bovis* (BCG-associated var.) or the H37R strain of Mtb (Miller et al., 2004).

Mtb infection results in inhibition of macrophage apoptosis as a means of increasing its survival (Velmurugan et al., 2007) (Figure 2). The serine/threonine protein kinase, PknE, interferes with the signaling pathways involved in apoptosis following NO stress (Jayakumar et al., 2008) and, thereby, modulates Mtb-induced macrophage survival.

Interleukin (IL)-1β, tumor necrosis factor (TNF)-α, and NOS2 are up regulated concomitantly in AM following exposure to Mtb (Bhatt and Salgame, 2007) (Figure 1). The production of NO by AMs in TB patients may have an auto-regulatory role which, through the activation of the transcription factor nuclear factor (NF)-κB, potentiates the generation of pro-inflammatory cytokines (Dlugovitzky et al., 2000; Kuo et al., 2000; Chan et al., 2001b). This hypothesis is supported by the presence of significantly higher levels of IFN-γ in milder cases of TB than in more advanced disease (Sahiratmadja et al., 2007). In contrast, patients with severe TB had greater levels of IL-12, transforming growth factor-β and TNF-α in comparison to those with less severe TB (Sahiratmadja et al., 2007). Moreover, nitrite levels were significantly increased in advanced TB patients compared with controls (Dlugovitzky et al., 2000). Over expression of the inhibitor of NF-κB, IκBα, confirmed that the IκBα kinase (IKK)–NF-κB signaling pathway enhanced IFN-γ- and Mtb lipoarabinomannan-induced NOS2 promoter activity and NO expression (Chan et al., 2001b).

Since Mtb affects one third of world's population and NOS2 seems to play an important role in growth of this bacilli (Raviglione et al., 1995), it is important to understand how genetic factors that may influence the susceptibility in disease of infected individuals. Thus, a combination of polymorphisms within the host NOS2 locus and the balance of NOS2- inducing or NOS2-inhibiting cytokines (MacMicking et al., 1997) may affect susceptibility to disease. In contrast, the expression of microbial genes that confer resistance to nitroxergic products (Nunoshiba et al., 1995; Hausladen et al., 1996; Nicholson et al., 1996) may regulate infectivity and disease latency.

## Mechanisms of granuloma formation and the role of NO

Granuloma formation following exposure to Mtb is correlated with strong inflammatory and protective responses. The expression of NOS2, NOS3 and nitrotyrosine (N-tyr) are all increased in the granuloma-associated inflammatory cells and in pneumonitis regions of human tuberculous lungs (Jung et al., 2013). In addition, granuloma-associated macrophages from untreated patients with pleuropulmonary and pulmonary TB demonstrate high levels of NOS2-mediated NO production and of N-tyr (Schön et al., 2004). The elevated expression of NOS isoforms and N-tyr is predominantly within AM, epithelioid macrophages and multinucleated giant cells (Choi et al., 2002) (Figure 2).

The progressive granulomatous response to TB can be tissue damaging and contribute to chronic infection, at least in immunocompetent hosts. NO can however, have a ying-yang effect on the clearance of infection and the inflammatory response depending upon the mycobacterial strain. IFN-γ and NOS2 knockout mice highlight the critical role of these mediators in protective immunity against Mtb (Cooper et al., 2002). Results suggest that they are important in the resolution of inflammation resulting from an increased lymphocytic response and can also decrease tissue damage as measured by granuloma regression (Cooper et al., 2002). However, during infection with *Mycobacterium* avium, which is less dependent upon IFN-γ and NO for preventing the growth of bacilli, a lack of NO results in a shift in the pattern of immunological response leading to increased bacterial clearance and enhanced the inflammatory response (Cooper et al., 2002). Thus, the effects of NO on mycobacterial growth and on the inflammatory and immune response are complex and strain-dependent.

## NO and drug resistant tuberculosis

A reduced ability of NO to kill disease-causing strains of Mtb was found to be associated with first-line anti-TB drug resistance (Idh et al., 2012). Earlier studies indicated that, certain strains of Mtb including *M. intracellulare* 31F093T, KUMS 9007 (Doi et al., 1993), *M. tuberculosis* CDC1551, CB3.3 (Firmani and Riley, 2002), *M. bovis, M. tuberculosis* 79499 (O'Brien et al., 1994), a C strain cluster defined by IS6110-based strain-typing (Friedman et al., 1997) and the genotypes G1, G2, S2, and U (Idh et al., 2012) can all to some extent resist killing by acidified nitrite, a RNS, generated *in vitro*.

The resistance of the CDC1551 and CB3.3 strains of Mtb to H2O2 and acidified sodium nitrite is significantly higher than that of other strains which may account for why these two species may be responsible for large outbreaks of TB (Firmani and Riley, 2002). In isoniazid-resistant strains, H2O2 susceptibility correlated well with the presence of small amounts of catalase but this does not account for low-virulence, isoniazid-sensitive, catalase-positive strains (Firmani and Riley, 2002).

Under physiological conditions of 10% rather than 21% oxygen, Mtb within infected primary human macrophages utilize nitrate and generating large quantities of nitrite (Cunningham-Bussel et al., 2013). Mtb lacking a functioning nitrate reductase, *narG*, are more susceptible to isoniazid and are more resistant to H2O2 and these phenotypes can be reversed by exogenous nitrite (Cunningham-Bussel et al., 2013). This suggests that nitrite production by Mtb under normal conditions may induce isonicotinic acid hydrazide (INH) insensitivity (Cunningham-Bussel et al., 2013).

This indicates that a common mechanism for both peroxide and RNS resistance may exist. Since the mechanism of action for anti-TB immunity is via the production of free radicals (Figure 1), strain variations in repair systems related to ROI and RNS, comparable to that seen in DNA repair, might be important (O'Brien et al., 1994).

Multi-drug-resistant tuberculosis (MDR) is a major threat to global health (Matteelli et al., 2007). There are currently two promising new drugs, the bicyclic nitroimidazoles, PA-824 and OPC-67683, which are currently undergoing human clinical trials (Li et al., 2008; Stop TB Initiative and World Health Organization, 2008). These agents are both active against actively replicating bacteria, as well as, bacteria that are non-replicating by virtue of hypoxia (Stover et al., 2000). Non-replicating cells are particularly difficult to eradicate and may be a major cause of relative treatment insensitivity and the need for long treatment periods (6–8 month) and disease relapses (Boshoff and Barry, 2005). Moreover, these non-replicating bacteria are thought to be associated with latent tuberculosis (Gomez and McKinney, 2004; Singh et al., 2008).

## Mechanisms of NO-mediated Mtb killing

The mechanism(s) by which pathogens such as Mtb suppress NO production are varied and have been recently elucidated. Initial studies indicated that IFN-γ-induced NOS2 expression was mediated via the transcription factor IFN regulatory factor-1 (Kamijo et al., 1994). More recent evidence indicates that although this process may not be critical for the control of early bacterial infection, it clearly plays a role in the granulomatous response (Cooper et al., 2000) (Figure 2).

The Proline-Proline-Glutamate (PPE) family of proteins are particularly abundant in pathogenic Mycobacterial species such as Mtb (Bhat et al., 2017). Recent evidence indicates that these proteins can act as repressive transcription factors in the nucleus of host macrophages to suppress NOS2 expression and thereby reduce the release of the anti-mycobacterial NO molecule (Bhat et al., 2017). In addition, soon after Mtb infection, macrophages produce IL-10 which induces the phosphorylation and activation of the transcription factor signal transducer and activator of transcription (STAT3) (Queval et al., 2016). STAT3 activation results in repression of NOS2 expression and reduced NO production allowing Mtb infection to occur. Importantly, intracellular Mtb replication is attenuated by the loss of STAT3 expression with the resultant increase in NOS2 (Queval et al., 2016).

However, alternative mechanisms for the control of Mtb infection have been proposed that center on the control of inflammation *per se* rather than on direct NO-induced inhibition of mycobacterial growth. Protective immunity to TB requires the release of IFN-γ from T-cells which induces host cell NOS2 expression and enhances NO production (Figure 1) (Mishra et al., 2013). NO prevents Mtb growth and the subsequent inflammatory response. NO can also directly modulate inflammation to impact upon Mtb growth and function (Mishra et al., 2017). NO acts to prevent the growth and immunopathology caused by TB via S-nitrosylation and inhibition of the Nod-like receptor (NLR) Family Pyrin Domain Containing 3 (NLRP3) inflammasome factor (Mishra et al., 2013). NLRP3 inhibition results in a reduction in IL-1β expression and in neutrophil recruitment. Neutrophilic inflammation generates a local niche that supports *M. tuberculosis* growth (Mishra et al., 2017). The presence of 12/15-lipoxygenase (ALOX12) products in cavitary tuberculosis lesions is correlated with airway neutrophilia and bacterial burden (Mishra et al., 2017). A genetic polymorphism associated with elevated ALOX12 expression is associated with an enhanced risk of tuberculosis. The data suggests that preventing the NLRP3/IL-1β/neutrophilic axis will prevent Mtb replication independent of NO (Mishra et al., 2017).

The importance of inflammation in the control of Mtb infection is further demonstrated by the ability of thymoquinone (TQ), an essential compound of *Nigella sativa* (black cumin) (Mikaili et al., 2013), to suppress Mtb-induced bacterial replication and inflammation in human and murine macrophage cell lines (Mahmud et al., 2017). Importantly, TQ acts despite markedly suppressing NOS2 expression and attenuating the host cell production of NO (Mahmud et al., 2017).

## Mtb protection against NO-mediated killing

In addition to the densely mycolylated cell wall, Mtb use other mechanisms to circumvent the host defense system and prevent killing by the host including those induced by pattern recognition receptors (PRRs) (Mortaz et al., 2017). PRRs are critically important in the host response to Mtb infection and their roles are summarized in several recent reviews (Mortaz et al., 2017). Full activation of murine macrophages depends upon IFN-γ, PRR activation, and/or TNF whereas vitamin D2 is required as a cofactor for maximal activation of human macrophages. This full activation results in enhanced expression of antimicrobial peptides/proteins (AMPs), such as cathelicidin and other antimicrobial moieties including ROS and RNS generation (Awuh and Flo, 2017). NO and ROS interact within the phagosome to generate highly reactive intermediates that destroy microbial membrane lipids, DNA, and thiol- and tyrosine residues by oxidation. NO also directly targets the iron sulfur clusters of bacterial enzymes (Awuh and Flo, 2017).

Mycobacteria have evolved over time to develop systems that reduce the antimicrobial activity of ROS (Awuh and Flo, 2017). For example, Mtb express many anti-oxidant enzymes such as superoxide dismutase, catalase, alkyl hydroperoxidase, and peroxiredoxins, to neutralize the free radicals generated by the host (Awuh and Flo, 2017). Furthermore, elevated expression of the Mtb protein, enhanced intracellular survival (Eis), increases intracellular Mtb survival. Eis has ROS-dependent effects on autophagy and inflammatory responses including the expression of TNFα and IL-6 to prevent cell death. Generally, Mtb can detect ROS/RNS-induced changes in the host environment and respond by producing proteins that limit the toxic effects of these changes (Awuh and Flo, 2017). This enables survival of Mtb in the nutrient-deficient, hypoxic and ROS/RNS-high environment present within granulomas (Awuh and Flo, 2017).

In addition, the Mtb proteasome protects the microbe from the damaging effects of NO and its derivatives. NO and RNI may modify or irreversibly damage Mtb proteins, possibly by nitrosylation, and this is counteracted by the removal of the damaged or modified proteins (Rhee et al., 2005). The key Mtb proteins involved in this protective response are the proteasomal accessory subunits proteasome accessory factor A (Paf) and Mycobaterium proteasomal ATPase (Mpa) (Wang et al., 2009). These proteins either recognize damaged proteins and deliver them to the proteasome or repair the damaged proteins. Mice challenged with Mpa or Paf mutant mycobacteria could combat infection because the mutants had reduced virulence (Ehrt and Schnappinger, 2009). Finally, the Mtb proteasome substrates, malonyl Co-A acyl carrier protein transacylase and ketopantoate hydroxymethyltransferase, are essential for Mtb pathogenesis. This suggests that targeting the Mtb proteasome may represent a novel anti-TB therapeutic approach (Darwin et al., 2003; Pearce et al., 2006).

## Diagnostic analysis of NO and NO metabolites

Gustafsson et al. (1991) first described the presence of NO in exhaled breath (Gustafsson et al., 1991). Thereafter, numerous reports showed that the concentration of exhaled NO is increased in patients with several lung diseases (Kharitonov, 2004). FeNO can be measured both in real-time (online) and off line (collected and then sent to a remotely located analyser) but it is now generally measured online by having the subject blow directly into the analyser (Kissoon et al., 2000; Olivieri et al., 2006). Interestingly, there is a significant difference in FeNO values between men and women; with a higher level in men (range 2.6–28.8 ppb) compared to women (range 1.6–21.5 ppb) at expiratory flows of 50 ml/s (Olivieri et al., 2006). The mechanisms underlying this difference may reflect an effect of estrogen on NOS2 expression but more research in this field is required (Olivieri et al., 2006). In addition, it is unclear whether there are differences in FeNO and NOS2 expression in alveolar macrophages in men and women with active pulmonary TB (Wang et al., 1998).

The urinary levels of the NO metabolites nitrite and nitrate are elevated in patients with active tuberculosis and these levels are reduced with anti-TB treatment (Chan et al., 1992). The endogenous generation of NO, as a defense mechanism against Mtb, is the most probable explanation for this (Chan et al., 1995; Nicholson et al., 1996). These two NO metabolites have frequently used as an indirect measurement of the production of NO *in vivo* (Anstey et al., 1996; Dykhuizen et al., 1996; Ellis et al., 1998; Sundqvist et al., 1998; Schön et al., 1999). In an interesting study that compared TB infected patients with and without HIV co-infection; the patients without HIV infection showed significantly higher amounts of FeNO (>25 ppb), compared to the patients with HIV and TB co-infection (Idh et al., 2008). In contrast, the amounts of urinary NO were greater in HIV/TB co-infected patients. FeNO or urinary NO levels did not significantly correlate with clinical signs, the grade of chest X-ray or inflammatory cytokine levels (Idh et al., 2008). In both HIV negative and HIV co-infected TB patients, there were low levels of FeNO compared to blood donors and household contacts. It would be an interesting topic for future studies to confirm whether low levels of FeNO could be used as a risk factor for acquiring TB (Idh et al., 2008). A corollary to this is that immunosuppressed patients, such as those with HIV, mount a less effective T-cell response to infection which may result in a reduced FeNO level (Idh et al., 2008).

The expression of NOS enzymes or levels of NO in various compartments may also represent a good biomarker for disease (Nicholson et al., 1996). BAL macrophages from patients with Mtb express higher levels of NOS2 mRNA and this has been linked to higher FeNO levels in the patient (Nicholson et al., 1996). Changes in serum levels of nitrites and nitrates as well as NOS2 activity in blood neutrophils may be another prognostic tool to predict the treatment outcome of TB infection (Butov et al., 2014).

## Therapeutic use of NO and NO-donors

NO plays an important role in the host defense against intracellular pathogens, but different cells in human body generate different amounts of NO. For example, murine macrophages produce sufficient levels of NO to act as a bactericidal effector molecule (Singh et al., 2008) and this may also occur in human macrophages (Fang and Vazquez-Torres, 2002). Many invading organisms compromise host macrophages by impairing host NOS2 activity resulting in reduced NO production. Decreased host NO production will, therefore, result in a more sustainable niche for host infection. It is posited therefore, that NO donors given to TB patients will be able to compensate for the lack of endogenous NO by the compromised macrophages (Seabra et al., 2015; Seabra and Duran, 2017). The importance of NO in the control of Mtb infection is also indicated by evidence in other species. NOS2 expression and NO production is extremely limited in macrophages of the European badger (*Meles meles*) and this may account for the species acting as a reservoir for the bovine tuberculosis (Bilham et al., 2017).

Further support of the importance of NO in the pathogenesis of TB is that these patients are deficient in both L-arginine, the NO precursor, and in vitamin D (Ralph et al., 2008). These analytes both have anti-TB and immunomodulatory actions against TB *in vitro* (Ralph et al., 2008). Furthermore, the levels of FeNO were significantly lower in patients with pulmonary TB than in controls, particularly in those with more severe disease, possibly reflecting reduced NO bioavailability (Ralph et al., 2013). Of interest, subjects whose FeNO levels were elevated or remain unchained after 2 months of anti-TB treatment had better mycobacterial clearance (Ralph et al., 2013).

Therefore, low molecular weight NO-donors should enhance Mtb killing and/or prevent intracellular replication. Indeed, phenylsulfonyl furoxan derivatives which are effective NO-donors have low micromolar efficacy against Mtb H37Rv (ATCC 27294) and a clinical isolate MDR-TB strain *in vitro* (Fernandes et al., 2016). Interestingly, clinical isolates of Mtb with reduced survival after exposure to the NO donor DETA/NO had a reduced response to first-line anti-TB drugs (Idh et al., 2012).

Altogether, this data suggests that increased NO delivered to the lung of patients with pulmonary TB might reduce infectivity and improve the response in patients with drug-resistant TB (Ralph et al., 2008). However, a 4-arm randomized, double-blind, placebo-controlled factorial trial in adults with smear-positive pulmonary TB in Indonesia, showed no clinical benefit of combined oral vitamin D and L-arginine over 8 weeks (NCT00677339)(Ralph et al., 2013). The failure to achieve an improved clinical outcome may reflect the inability of L-arginine to enhance NO production in the airways as reflected by a failure to increase FeNO in patients on the active treatment. This highlights the need to develop better, more effective NO-donor platforms to deliver therapeutic doses of NO to the correct biological site and several approaches are being utilized toward this end (Seabra and Duran, 2017). The therapeutic use of gaseous NO itself may not be practical due to the cumbersome nature of the machine, the expertise intensive procedure and the long duration of intermittent exposure required to show efficacy would be difficult to implement on ambulatory patients. In addition, NO may cause tissue injury within the lung and long-term exposure to NO may causes cardiovascular and other pharmacological side effects and should not be given to patients diagnosed with end stage renal disease or severe left ventricular dysfunction for example. Therefore, inhaled NO-donors which deliver high levels of intracellular NO to macrophages may give better results (Verma et al., 2012).

INH is an important anti-TB agent and can produce NO following oxidation in cells infected by Mtb (Long et al., 2005). The importance of this process in INH function was emphasized by the ability of NO scavengers to attenuate the anti-mycobacterial activity of INH in cell culture (Long et al., 2005). Furthermore, inhaled NO (80 ppm) was safely administered to patients with pulmonary TB (Long et al., 2005) but this had no effect on the mycobacteriologic response achieved with conventional therapy. There is also a need for more studies to determine whether inhaled NO, delivered over the first 48 h, has a significant early bactericidal activity as measured by a reduction in the rate of decline of sputum Mtb numbers particularly in patients with MDR or drug-intolerant disease (Long et al., 2005).

The design of NO-donor moieties has developed significantly over the past decade although there are still major steps required to translate these *in vitro* technologies into clinical drugs (Seabra et al., 2015). There is an increasing range of these NO-donors that have extended beyond traditional low molecular weight NO donors such as S-nitrothiols (RSNOs) and NONOates toward novel biomaterials including NO-releasing nanomaterials such as polymeric nanoparticles, dendrimers and liposomes/micelles. These NO-releasing nano-materials may enable the correct spatio-temporal production of NO within the airway macrophages to target Mtb infection (Seabra et al., 2015).

Polymeric nanoparticles are biocompatible whilst the dendrimer scaffold has the ability to store large amounts of NO, due to their highly branched structure. However, dendrimers are difficult to manufacture and the process involves toxic organic solvents. In addition, polymeric micelles have a low thermodynamic stability in biological fluids resulting in doses which are usually too low to be effective (Seabra et al., 2015).

Improved medicinal, computational and click chemistry approaches should result in the development of more heat-stable drugs with enhanced sustained NO release profiles preferably at the disease site. Drug efficacy may be improved for pulmonary TB by using inhaled delivery. As with all new drugs, there is a need to determine the effect of chronic dosing *in vivo* matched to improved biodistribution and pharmacokinetics/pharmacodynamics for each compound. With improved features, it is likely that the scale-up costs for development will be markedly reduced (Seabra et al., 2015).

The identification of volatile organic compound (VOC) signatures in response to active infection with Mtb in macaques raises the possibility of the rapid determination of clinical efficacy of treatments such as NO-donors. Three compounds, dodecane, hexylcyclohexane, and tridecane, may be the most promising as they are also seen in humans infected with Mtb (Mellors et al., 2017). Thus, measures of FeNO or of VOCs in exhaled breath may enable dosing to be calibrated according to the dose required (Mellors et al., 2017). It is likely that these novel NO-donating polymeric nanomaterials will be used in concert with current low molecular weight NO donors to achieve maximal therapeutic effect.

As highlighted above, IFN-γ is important in the human immune response to Mtb. Delivery of aerosolized IFN-γ given in conjunction with standard anti-TB therapy enhanced expression of NOS2 and IFN-inducible protein 10 (IP-10) mRNA expression in AM of TB patients (Raju et al., 2004). Despite other studies providing strong evidence for *in vitro* anti-mycobacterial activity of IFN-γ in mouse macrophages (Rook et al., 1986; Flesch and Kaufmann, 1987; Denis, 1991), another study reported that IFN-γ is relatively ineffective in restricting intracellular Mtb growth in human macrophages. The anti-mycobacterial effect of IFN-γ was enhanced by adding retinoic acid and vitamin D3 (Douvas et al., 1985; Cholo et al., 2005). However, due to the potential of side-effects and the costs it is unlikely that this approach will be pursued as an anti-TB therapy.

## Conclusion

In conclusion, there have been significant increases in our understanding of the mechanisms by which NO regulates Mtb growth and emphasize this as a potential target for anti-TB therapy. Indeed, NO-donating drugs have therapeutic potential in a number of human diseases including TB (Rigas and Williams, 2008). Analysis of the effect of these novel agents, and of other modifiers of Mtb proliferation including immunomodulators and Mtb nitrate reductase inhibitors, should be undertaken in primary human cells under physiological conditions. It is also important that sufficient NO is delivered to the target cell within the airway and that the effect can be monitored effectively to provide a rapid readout of drug action. Thus, measurements of FeNO or of specific VOCs are essential to monitor drug efficacy and enable variable dosing to minimize any potential side-effect issues. Novel NO-donors, particularly polymeric nanoparticles ideally delivered by the inhaled route for pulmonary TB, show promise and may be improved by structure-based design to produce agent(s) that not only treat but also have the potential to cure active and latent tuberculosis (Singh et al., 2008). However, the importance of inflammation in Mtb pathophysiology must also be considered when treating these patients. Overall, increased understanding the role of NO in Mtb pathophysiology has provided great insight into many aspects of disease mechanisms and elucidated potential novel treatments.

## Author contributions

HJ, EM, and ZP wrote the original draft of manuscript. GF and MeM helped with literature collating and referencing. MiM, IMA, and JG revised and edited the manuscript.

### Conflict of interest statement

The authors declare that the research was conducted in the absence of any commercial or financial relationships that could be construed as a potential conflict of interest.
